# MS4 - Multi-Scale Selector of Sequence Signatures: An alignment-free method for classification of biological sequences

**DOI:** 10.1186/1471-2105-11-406

**Published:** 2010-07-30

**Authors:** Eduardo Corel, Florian Pitschi, Ivan Laprevotte, Gilles Grasseau, Gilles Didier, Claudine Devauchelle

**Affiliations:** 1 Georg-August-Universität, Institut für Mikrobiologie und Genetik, Goldschmidtstraβe 1, 37077 Göttingen, Germany; 2 Partner Institute for Computational Biology, CAS-MPG, 320 Yue Yang Rd, 200031 Shanghai, China; 3 Laboratoire Statistique et Génome (LSG), CNRS UMR 8071, INRA 1152, Université d'Evry, Tour Evry2, Place des Terrasses, 91034 Evry Cedex, France; 4 Institut de Mathématiques de Luminy, UMR 6206, Luminy, Marseille, France

## Abstract

**Background:**

While multiple alignment is the first step of usual classification schemes for biological sequences, alignment-free methods are being increasingly used as alternatives when multiple alignments fail. Subword-based combinatorial methods are popular for their low algorithmic complexity (suffix trees ...) or exhaustivity (motif search), in general with fixed length word and/or number of mismatches. We developed previously a method to detect local similarities (the *N*-local decoding) based on the occurrences of repeated subwords of fixed length, which does not impose a fixed number of mismatches. The resulting similarities are, for some "good" values of *N*, sufficiently relevant to form the basis of a reliable alignment-free classification. The aim of this paper is to develop a method that uses the similarities detected by *N*-local decoding while not imposing a fixed value of *N*. We present a procedure that selects for every position in the sequences an adaptive value of *N*, and we implement it as the MS4 classification tool.

**Results:**

Among the equivalence classes produced by the *N*-local decodings for all *N*, we select a (relatively) small number of "relevant" classes corresponding to variable length subwords that carry enough information to perform the classification. The parameter *N*, for which correct values are data-dependent and thus hard to guess, is here replaced by the average repetitivity *κ *of the sequences. We show that our approach yields classifications of several sets of HIV/SIV sequences that agree with the accepted taxonomy, even on usually discarded repetitive regions (like the non-coding part of LTR).

**Conclusions:**

The method MS4 satisfactorily classifies a set of sequences that are notoriously hard to align. This suggests that our approach forms the basis of a reliable alignment-free classification tool. The only parameter *κ *of MS4 seems to give reasonable results even for its default value, which can be a great advantage for sequence sets for which little information is available.

## Background

The classification of biological sequences is one of the fundamental tasks of bioinformatics, and faces special challenges in the genomic and post-genomic era. While it is a classical paradigm to base it on an initial multiple alignment of the sequences, a current trend is to provide alignment-free classification methods (subword-based [1], kernel-based [2], composition vector-based [3,4]...), in order to tackle datasets that cannot be amenable to multiple sequence alignment (MSA) methods. Approaches based on *k*-mers have also been used for more than a decade to detect anchoring zones for whole genome alignments [5-8].

In this paper, we describe a method for the alignment-free classification of families of nucleic or protein sequences (composed of a few hundreds of members). Our aim is to rapidly detect similarity segments shared by these sequences without having to consider the order in which they occur inside the sequences. Our approach allows us to take into account shuffled domains as well as repeated segments.

The local similarity detection uses a previously described method called *N*-local decoding [9]. The basic principle of the *N*-local decoding is to rely on the occurrences of similar substrings in sequences to cluster together positions in the sequences. More precisely, two positions in the considered sequences (that we will call "sites" for short) are *directly related *when they occur at the same position in two equal substrings of fixed length *N*. The *N*-local decoding clusters together all *indirectly *related sites, that is, sites related by a chain of direct relations. This results in a *partition *of the set of sites. For each subset of clustered sites (an *equivalence class *or simply *class*), the segments of length 2*N *- 1 which are centered on the sites exhibit local similarities. Although it is based on exact matches, the indirect relation scheme results in the inclusion of an *a priori *unknown number of mismatches.

We have previously used successfully this *k*-mer based method for alignment-free classification [10], without being able to solve the delicate problem of tuning the parameter *N*. In the present paper, we tackle this problem by developing a procedure to select among all the segments of similarity detected by *N*-local decoding for all *N*, a subset on which to base the classification. We call this alignment-free classification method MS4, for Multi-Scale Selector of Sequence Signatures.

The *N*-local decoding has been efficiently implemented using suffix trees. Like in any *k*-mer based approach, there is no sensible criterion to fix a value of the parameter *N*. Here, we follow how the partition of sites varies with the parameter *N*. When *N *increases, site classes tend to split into several subclasses, while for too low values of *N*, classes tend to group sites that do not share any detectable similarity. MS4 attempts to select among all these classes of sites those that correspond to relevant homologous segments. More precisely, MS4 selects for a given site the smallest *N *such that the average number of occurrences per sequence of the equivalence class of this site is smaller than a given threshold *κ*. The resulting values of *N *are different for different sites, and adapt to the context of appearence of the site among the studied set of sequences. The parameter *κ*, unlike *N*, has a sensible global interpretation, and can be tuned to a value reflecting the maximum number of repetitions in the sequences. Finally, the classes selected by MS4 are used to compute a dissimilarity matrix on which the classification is based (using the NeighborNet option of SplitsTree [11,12]).

In this paper, we describe the implementation of the MS4 classification tool, which is accessible via a Web-based interface. We also give a validation on some real biological data that are not so easy to classify: MS4 is illustrated on several families of HIV/SIV sequences. These sets have already been classified by us with the help of *N*-local decoding method [13], and it was shown that the *N*-local decoding classes correspond to segments of homology for these sequences [10]. The results obtained in [10] were in good agreement with the accepted classification [14,15], for several values of *N*. These "good" values are however data-dependent and hard to guess. The approach described in this paper replaces this parameter with the more intuitive parameter *κ*.

Our present results show that MS4 gives correct classifications on coding and non-coding regions of HIV/SIV. Moreover the results are robust with respect to the variations of the parameter *κ*. In fact, even on sequences containing repetitions (like the non-coding regions of the HIV/SIV LTR), the choice of *κ *= 1 gives satisfying results. Therefore, MS4 may be expected to give reasonable results for this default value for *κ *when no other information on the sequences is available.

## Methods

As mentioned in the Background section, we use the *N*-local decoding (NLD) in order to produce partitions of the set of all sites in the sequences under study [9]. A short recapitulation of NLD is found here. The central part of this paper is the introduction of an object that describes the embedding of successive partitions as *N *increases. It turns out that this object is a tree. The tree structure is essential, because it provides a criterion for choosing "relevant" partitions of sites, which may occur at several values of *N*. We use the chosen classes to construct a dissimilarity matrix between sequences (taxa). This matrix becomes then the input for standard tree construction methods (SplitsTree4 [11,12] in our case).

### *N*-Local Decoding

We consider a collection ***S ***of sequences s over a finite alphabet . The *site space *consists of all pairs *σ *= (*s, p*) where *s *is a sequence, and *p *a position in it. This set is(1)

where ℓ (*s*) is the length of sequence *s*. The NLD procedure starts with a collection of sequences and with an integer *N *≥ 1. It consists of two steps:

1. To every site *σ *in ∑, associate a neighborhood of length 2*N *- 1, consisting of *σ *and of *N *- 1 sites on each side of *σ *(neighborhoods that are too near the beginning or the end of a sequence are accordingly truncated, but this case will not be considered for simplicity's sake in the rest of the description). This neighborhood carries a word *W *of length 2*N *- 1. We consider all subwords *w *of length *N *of this word *W*. They can be "identified" by their *position relative to σ*, i.e. the index of the beginning of *w *inside *W*. The subword *w *of *W *at relative position *i *will be denoted by *w_i_*. Given two sites *σ *and *σ'*, we say that they are *directly related *if there exists an *i *such that the subword *w_i _*of *W *is identical to the subword  of *W' . *If two sites *σ, σ' *are directly related, we write *σ* ≃_*N *_*σ'*.

2. We define the equivalence relation ~_*N *_as the transitive closure of ≃_*N *_. In other words, we say that *σ*_1 _~_*N *_*σ*_2 _if there is a chain of directly related sites connecting *σ*_1 _and *σ*_2_.

**Figure 1:**
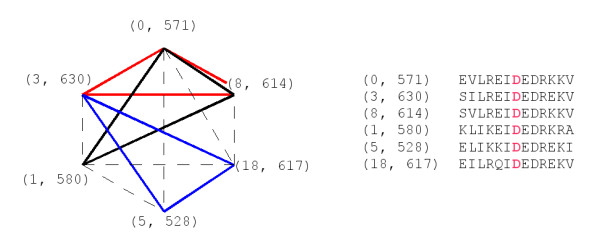
**NLD illustration**. Graphical representation of relatedness within an NLD class, with *N *= 7. For each one of six sites, the word occupying its neighborhood is shown on the right hand of the picture. Directly related sites are connected by solid lines: each color corresponds to (at least) one word of length 7 shared by two neighborhoods. Broken lines connect sites that are connected but not directly connected.

We illustrate this on an example (Fig. 1). We consider here a set of protein sequences, and examine one of the equivalence classes obtained by *N*-local decoding with *N *= 7. This class consists of 6 sites. The first site is described by the pair (0,571): this means that it lies at position 571 of the sequence number "0", and similarly for the other five sites. Since *N *= 7, the neighborhoods around these sites are of length 2*N *- 1 = 13. The words in these neighborhoods are shown on the picture, with the central letter displayed in red.

Directly related sites are connected by solid lines. For instance, the sites (0, 571); (3, 630) and (8, 614) share the word LREIDED starting at the third position of their environment. The sites that are related (but not directly related) are connected by broken lines. For instance, the sites (1, 580) and (5, 528) are connected by the chain (1, 580) → (0, 571) → (3, 630) → (5, 528). The fact that every site is connected to every other site means that this set of sites is a class.

### The Partition Tree

A recurring problem of *N*-mer-based methods is that there does not seem to be a good criterion to tune this parameter *N *to an acceptable value. There is moreover no real reason to believe that a single "optimal" value will always be meaningful, since the similarity between sequences can depend very much on the position of neighborhoods in sequences.

In the case of *N*-local decoding, we combine the different equivalence classes for various values of *N *by introducing a new construction, the *partition tree*, which encodes the way in which equivalence classes for successive values of *N *are related. This tree will allow us to choose a set of "relevant" NLD-classes. Let ℰ*^N ^*be the partition of ∑ induced by ~*_N_*.

**Lemma 1**. *For all N *≥ 0, *the partitions *ℰ^*N *^*satisfy *ℰ^*N*+1 ^⊂ℰ^*N*^.

*Proof*. Compare the partitions of ∑ produced by ~_(*N*+1) _with the partitions produced by ~*_N_*. If any two sites *σ*_1 _and *σ*_2 _are ~_(*N*+1)_-equivalent, we have to show that they are ~*_N_*-equivalent. Notice that *σ*_(*N*+1) _equivalence is reduced to a set of direct ≃_(*N*+1) _relations, and that *σ*_1 _≃_(*N*+1) _σ_2 _implies trivially *σ*_1 _≃*_N _σ*_2_. If two neighborhoods share a word of length *N *+ 1 at a given relative position, they also share words of length *N *at the same relative positions.

This simple lemma is crucial, and corresponds to the intuitive idea that it is harder to lump together big words than small words. We are now ready to define the partition tree.

**Definition 1**. *For N > 0, denote by *ℰ*^N ^the set of equivalence classes defined by the relation ~_N_. Letting *ℰ*^0 ^= *{}*(which will correspond to the root of the tree), we can encode the set V *= ∪_*i *≥ 0 _ℰ^*i *^*of equivalence classes for different values of N into the *partition tree **P ***= (V, E***^P^***), defined by*

**Figure 2:**
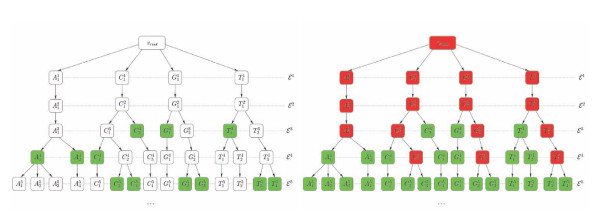
**Selection of relevant classes**. Selection of relevant classes in a partition tree. On the right, the green nodes satisfy *κ *= 1 while the red ones do not. On the left, only the relevant classes are shown.

**Figure 3:**
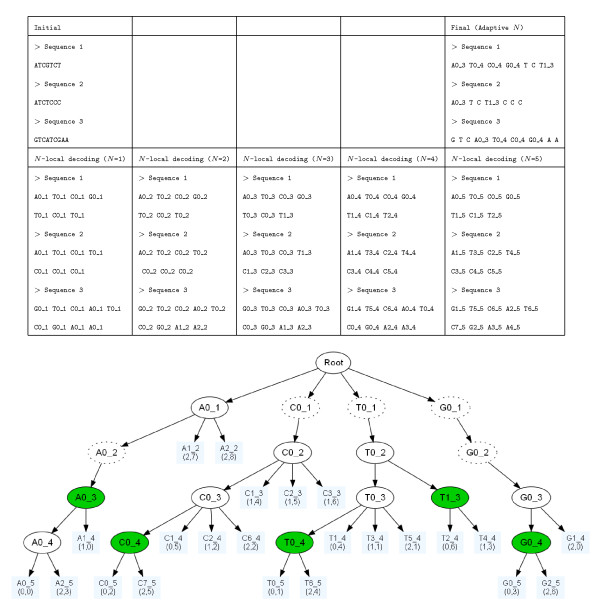
**Didactic example**. Toy example of Multi-Scale Selector of Sequence Signatures (MS4 selection of classes). On the first row (top) we see the input sequences and the output of eligible classes (MS4 classes). The second row shows the NLD re-writing from *N *= 1 to 5. The partition tree constructed on the basis of the re-writing is shown on the lower part of the picture. The leaves correspond to classes that contain a single site (singletons). The dotted nodes should normally disappear from the compacted tree, and are only shown for clarity's sake. The eligible classes are colored in green. Nodes are labelled with identifiers like C0_3  where C0 is an arbitrary class identifier and 3 the value of *N*.

In other words: the vertices of **P **are all the equivalence classes that correspond to ~*_N _*for all values of *N*. The edges are drawn between pairs of classes that correspond to successive values of *N *and such that one is a subset of the other. By the above lemma, any two sites that are (*N *+ 1)-equivalent are also *N*-equivalent. On the other hand two sites that are *N*-equivalent are not necessarily (*N *+ 1)-equivalent. In other words, the *N*-classes split as *N *increases. The edges are drawn precisely between any *N*-class *C *and all the (*N *+ 1)-classes into which *C *splits. From this definition, it is clear that any vertex of **P** has at most one ancestor, *i.e*. that **P **is a tree. Finally, for memory saving purposes, all valency 2 nodes are suppressed from **P **(resulting in the *compacted *partition tree). Examples of partition trees are given in Fig. 2 and Fig. 3.

### A choice of classes

When we examine *N*-equivalence classes for all possible *N*, we face a deluge of information, moreover altogether redundant. We shall now use the tree of partitions to alleviate this problem. Given any set *C *of sites, we can define the *size *of *C *as the number of sites in *C *and the spread of *C *as the number of sequences which contain at least one element of *C*. Define *κ*(*C*) as the ratio between the size and the spread of *C *as follows.(2)

For a given value *κ *≥ 1, the condition *κ *(*C*) ≤ *κ *means that the average number of occurrences of class *C *per sequence where it occurs is less or equal than *κ*. In particular, *κ *(*C*) = 1 means that no sequence contains more than one element of *C *(of course we take here *C *to be an NLD-class). We call the parameter *κ *the *maximum average repetitivity*. We use this parameter to select nodes in the partition tree that satisfy *κ *(*C*) ≤ *κ*.

This condition is not sufficient to make these classes relevant (see an example in Fig. 2). Indeed, the bottom of the partition tree is occupied by classes corresponding to large *N*, which occur in only one sequence. Such classes are of no interest. In order to find relevant classes, we have to "climb upward" (towards smaller values of *N*). Since any vertex of a tree has only one ancestor, the following definition does make sense.

**Definition 2**. *An NLD class C will be called κ-*relevant*, if it satisfies κ (C) ≤ κ, while its ancestor does not*.

The MS4 method consists in choosing all relevant classes in a set of sequences, and ignoring the others. The algorithm describing the implementation of MS4 is given in section Appendix. An explicit toy example on which we can see both the *N*-local decoding and the selection of relevant classes at work for *κ *= 1 is shown in Fig. 3.

### The Dissimilarity matrix

**Figure 4:**
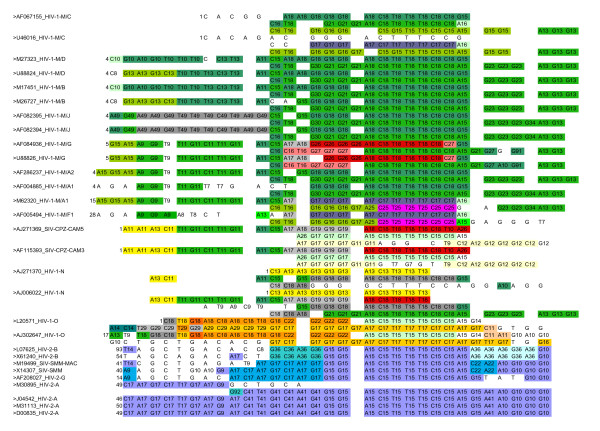
**Example of similarity blocks found by MS4 in the non-coding LTR sequences**. Part of the alignment from 29 out of the 43 non-coding LTR sequences centered on the NF*κ*B binding site. The complete alignment of the 43 sequences is shown in Additional Files 8 and 9. The alignment is focused on the transcription factor NF*κ*B binding site (GGGACTTTCC[A|G]) and its flanking regions. The names of sequences are indicated with their accession number in Los Alamos HIV sequence databank. The sequence are regrouped according to their phylogeny. The position of the first letter of the displayed region is given on the left. The letters are rewritten by applying the MS4 method to the whole non coding LTR sequences. As seen in Additional File 8, the complete MS4 identifier is constructed as follows: e.g. C24_8 (class C24 for a N value of 8). Identical recoded letters that are in the same columns are displayed in the same colour. The MS4 identifier has been simplified as follows: we have just indicated the letter and the value of N. Therefore it can be that two different MS4 classes that lie on the same column, with the same letter and the same N value are only distinguished by their colour (e.g. A18 and also T18 HIV-1-M/G, that are red or green). The two or more repeated segments of the same sequence are put one under the other. Therefore the sequences are often written on several lines to highlight similarities between sequences and inside sequences. Most often the similarity blocks are aligned and the great majority of identical indexed letters are on only one column. Some colored letters are unique because only 29 sequences (out of 43) are displayed on this figure.

At the end of the MS4 procedure, each sequence can be rewritten, by replacing the letter originally found at a given site by the identifier of the relevant MS4-class to which the site belongs (e.g. Fig. 4). We use the number of MS4 classes shared by 2 sequences to define a similarity index in a similar way as described in [10]. This measure is closely related to the percentage of identity classically used for sequence comparison.

Given any two sequences *seq_i _*and *seq_j_*, we compute a number *d_ij _*as follows. For a class *c*, let *n_i_*(*c*) be the number of occurrences of *c *in *seq_i_*. Denote by *C_ij _*the set of relevant classes that have representatives both in *seq_i _*and *seq_j_*. Since the two sequences can contain a different number of occurrences, we put . Let ℓ be the minimum of the lengths of *seq_i _*and *seq_j _*. We define then a dissimilarity *d_ij _*by(3)

In fact, *n_ij _*is the sum of local similarities shared by 2 sequences. Any exact common word of length *M *corresponds to *M *common MS4 classes (e.g. Fig. 4).

**Figure 5:**
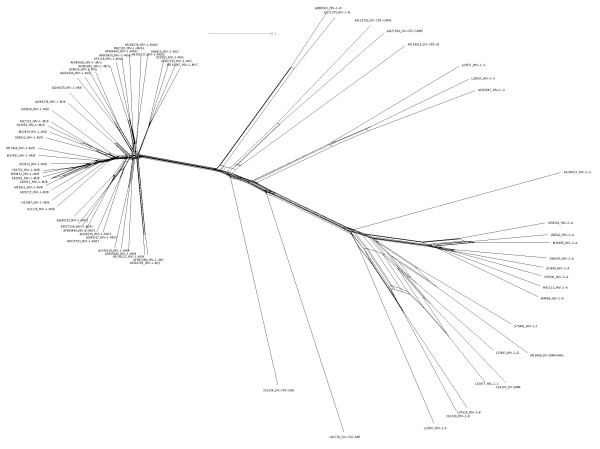
**Network from the HIV/SIV genomes**. The split-network obtained from 70 HIV/SIV genome sequences (dissimilarity matrix calculated by MS4). The sequences names are written as follows: their GenBank accession numbers, followed by their nomenclature names [15].

**Figure 6:**
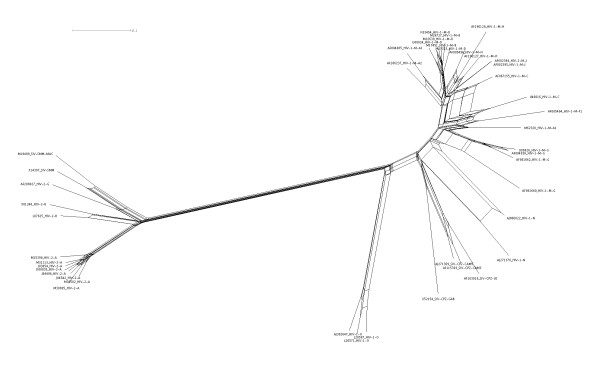
**Network from the non-coding LTR sequences**. The split-network obtained from 43 HIV/SIV non-coding parts of LTR nucleotide sequences (distance matrix calculated by MS4 for *κ *= 1 and *N *varying from 2 to 100). M15390 corresponds to the HIV-2-A ROD isolate just as X05291 for Fig.5. Sequence names follow the same rule as in Fig.5.

When *κ *= 1, *n_ij _*is simply the number of relevant classes having representatives in both *seq_i _*and *seq_j _*. This dissimilarity matrix is used as input in NeighborNet of SplitsTree4 [11,12] to produce the split networks displayed in Fig. 5 and Fig. 6.

## Results and Discussion

### MS4-classification of complete HIV/SIV genomes

We have applied the MS4-method, followed by a computation of the dissimilarity matrix (see section Methods), and the construction of a split-network (with the option NeighborNet [12] of SplitsTree4 [11]) to a family of 70 HIV/SIV genomes. The input for the calculation of the dissimilarity matrix consists of the classes selected by MS4 with *κ *= 1, for values of *N *between 2 and 60. We use here the same 70 non-recombinant HIV (Human immunodeficiency virus)/SIV (Simian immunodeficiency virus) nucleotide sequences that we studied previously in [10] by using the *N*-local decoding method. These sequences include four incomplete (*gag*) sequences (HIV-2 subtype C, D, E, F). These short sequences are subtyped in the sequence databases, so they appear to have kept subtyping signals that are in the complete genome sequences. The 66 complete sequences range in length from 8555 to 11443 nucleotides. All these sequences can be retrieved from the Los Alamos HIV sequence database [16] (their accession numbers are given in Fig. 5). The accepted groups are as follows:

1. HIV-1 group M (subtypes A-D, F-H, J, K; A is split into A1 and A2, and F is divided into F1 and F2),

2. HIV-1 group N,

3. HIV-1 group O,

4. HIV-2 groups A, B, G,

5. SIV-CPZ (chimpanzee)

6. SIV-SMM (sooty mangabey)

We produce a network by application of SplitsTree4 on the basis of a dissimilarity matrix given by the MS4 method. Fig. 5 shows the network obtained by our calculation. The network is quite tree-like. The two types of HIV are clearly distinguished: HIV-1 is closer to SIV-CPZ and HIV-2 is closer to SIV-SMM. The HIV-1 group M, on the left, is clearly separated from the rest. The nine subtypes of HIV-1 group M (major) cluster distinctly, with sub-subtypes significantly more closely related to each other (A1 and A2, F1 and F2, B and D that should be regarded as sub-subtypes [14,15]). Subtype K is more distant from sub-subtypes F1 and F2 than these are from each other, but closer to them that to other subtypes. The HIV-1 group N intercalates between HIV-1-M and SIV-CPZ (-CAM3, -CAM5, -GAB, and -US). The HIV-1 group O is intercalated between these CPZ and CPZ-ANT that is the borderline in the HIV-1/SIV-CPZ lineages. HIV-2 groups also form clear clusters, respectively, including C, D, E, and F that cover about half of the *gag *region.

Within the HIV-2 viruses, notice that the HIV-2 area, with the exception of the groups A and G, is less tree-like than the rest. From the aspect of the network, it seems that HIV-2-C tends to cluster both with HIV-2-B and with SIV-SMM. Another example is SIV-SMM-MAC which tend to group with both HIV-2-F and with HIV-2-D. Notice that the sequences HIV-2-C, HIV-2-D and HIV-2-F are short.

These groupings, which were obtained without alignments and without parameters, agree with accepted classifications.

In our previous paper, we varied the parameter *N *and we selected values of *N *that agree with existing knowledge; it turned out that correct tree topologies were found for *N *in the range from 13 to 35. The fact that the same groupings were found by the MS4 method with no other input than the sequences themselves gives us some confidence in the validity of this approach.

#### HIV/SIV sequences from the Compendium 2000

We have also calculated a split network from the 46 HIV/SIV complete nucleotide sequences of the Compendium 2000 (HIV-1/HIV-2/SIV Complete Genomes), and compared it with a tree available at [17]. The result of our calculation is tree-like, and agrees with the topology of the Compendium tree (Additional File 1).

#### Major genes of HIV/SIV

The major genes (*gag, pol, env*) of the HIV/SIV sequences (see above) were also tested.

1. For *gag *we have 70 sequences: 66 complete sequences (1473 to 1569 nucleotides in length) and 4 partial sequences covering about half the *gag *regions (771-781 nt).

2. For *pol *: we have 66 complete sequences (2993-3360 nt).

3. For *env *: we have 66 complete sequences (2499-2658 nt).

The regions *pol *and *env *were unavailable for the 4 HIV-2 groups C-F. The trees obtained for *gag*, *pol *and *env *give a good classification and the same description can be done for them as that detailed above for the 70 complete sequences (Additional Files 2, 3, 4).

### MS4-classification of short sequences: *nef *and non-coding LTR sequences

#### Non-coding LTR

In order to test our method, we have also looked at parts of the HIV/SIV genomes that are notoriously hard to align due to inner repetitions in the sequences. One of them (retrieved from 43 of the 70 sequences) covers the non-coding part of long terminal repeat (complete non-coding LTR region or at least its portion including the polyadenylation signal AATAAA). The lengths of this part range from 211 to 328 nt in the HIV-1/SIV-CPZ subset, and 433 to 508 nt in the HIV-2/SIV-SMM subset. These short non-coding segments contain many duplications/insertions/deletions that make them difficult for traditional alignment-based phylogenic studies.

The network obtained (Fig. 6) shows again a clear separation between HIV-1 and HIV-2, even though it was constructed with short and "difficult" subsequences. It is less treelike than the network obtained from the complete sequences, which is not surprising. The comparison between Fig. 5 and Fig. 6 show several features which may require further investigation: While the complete genomes produce very strong grouping of the sub-types HIV-1-M, the non-coding LTR show several discrepancies for these sub-types. The clustering of HIV-2 (and their groups), SIV-SMM, HIV-1-O, SIV-CPZ and HIV-1-M is correct. The network (Fig. 6) is similar to the tree in our previous paper [10].

It is interesting to notice that the two HIV-1-N are not very clearly grouped together. The sequence AJ271370_HIV-1-N is grouped both with the chimpanzee group (SIV-CPZ) and with AJ006022_HIV-1-N. On the other hand, AJ006022_HIV-1-N tends to group both with the other HIV-1-N and with AF061640_HIV-1-M-G (but less clearly). In the Neighbor Joining tree of [10], the two HIV-1-N are grouped together with a bootstrap value of 95% and connected with the group SIV-CPZ with bootstrap value of only 55%.

Even though our results show the difficulties of treating the non-coding part of LTR, it should be stressed that our method says something about these sequences. By contrast, these sequences are not tractable by standard alignment-based methods [10].

The featured sequences are reputedly hard to align, because they exhibit several repeated segments. MS4, used together with SplitsTree4, gives relevant results on these data that are usually set aside for the typing and subtyping of HIV-SIV, for lack of sufficient phylogenetic signal. This observation was already present in our previous study which used only the *N*-local decoding method. In this previous study, we proceeded to the careful - and tedious - scrutiny of several trees, resulting from the NLD method for various values of the parameter *N*. We showed that, for the non coding LTR sequences, the best tree (best fitting the reference classification) was obtained for the value *N *= 11. The splits networks that are obtained by MS4, or by NLD for *N *= 11 (Additional File 5), are similar and yield correct groupings of the non-coding LTR. One only notes a discrepancy inside group M, NLD giving a better clustering of the A subtypes, while MS4 groups H subtypes better.

It should be noticed that when we have here varied the maximum average repetitivity *κ *from 1.0 to 10.0 (by step of 0.5), the obtained classifications turned out to be remarkably robust to this variation (e.g. Additional Files 6 and 7).

#### NFkB region

We focus now on the non-coding region of LTR, to show how MS4 deals with repetitions in the sequences. The fig. 4 and the figures in Additional Files 8 and 9, show the binding site of the transcription factor NF*κ*B and its flanking regions [10]. This site is characterised by the signature GGGACTTTCC[A|G], which is present one or two times in the non-coding region of the LTR of HIV/SIV genomes (one or two additional imperfect copies may exist).

It clearly appears that, although the parameter *κ *is here set to 1, this zone contains relevant classes over the whole repeated region. Each repeated motif of the NF*κ*B pattern is identified by a different set of MS4-classes corresponding to *N *larger than the length of the repeated motif. Fig. 4 illustrates how the MS4-classes on this repetitive region participate to the overall MS4 classification. We clearly distinguish the HIV-1-N group which has some similarity with SIV-CPZ, the group HIV-1-O, and the group HIV-1-M in which we can distinguish *e.g*. the subtypes HIV-1-M/G, C and J. The HIV-2 sequences are clearly separated into three groups A, B and G which show similarities with SIV-MM. This example illustrates the facts that (a) Repeated segments are taken into account by the MS4 method, even for *κ *= 1 (which corresponds to one repetition of a class per sequence) and (b) each repeated segment participates in the classification of our set of sequences. Fig. 4 also illustrates the way that the re-writing of sequences in terms of MS4-classes defines the dissimilarity between sequences (See Eq.3). For instance, in the sequences HIV-1-M/J, a class, such as 'A49', corresponds to an exact word of length 49 shared by the two sequences. These classes correspond to the value *N *= 49 when the similarity concerns only 2 sequences (this is a straightforward exact match) but a smaller *N *when it is shared by more than 2 sequences (most often *N *= 18 for the binding site of NF*κ*B).

#### The nef sequences

We have also studied the 66 *nef *sequences (292-783 nt). The classification by MS4 is correct except for a few discrepancies (that have already been described in [10]): in the group HIV-1-M, sub-subtypes F1 and F2 mix together, and the position of subtype K is uncertain between F1/F2 and J (Additional File 10). In both cases (non coding LTR and *nef*) that we just saw, it is obvious that a full classification is not possible due to conflicting signals, and it is necessary to find homologous sites on a multiple alignment (as we did for LTR with *N*-local decoding in [18]).

**Figure 7:**
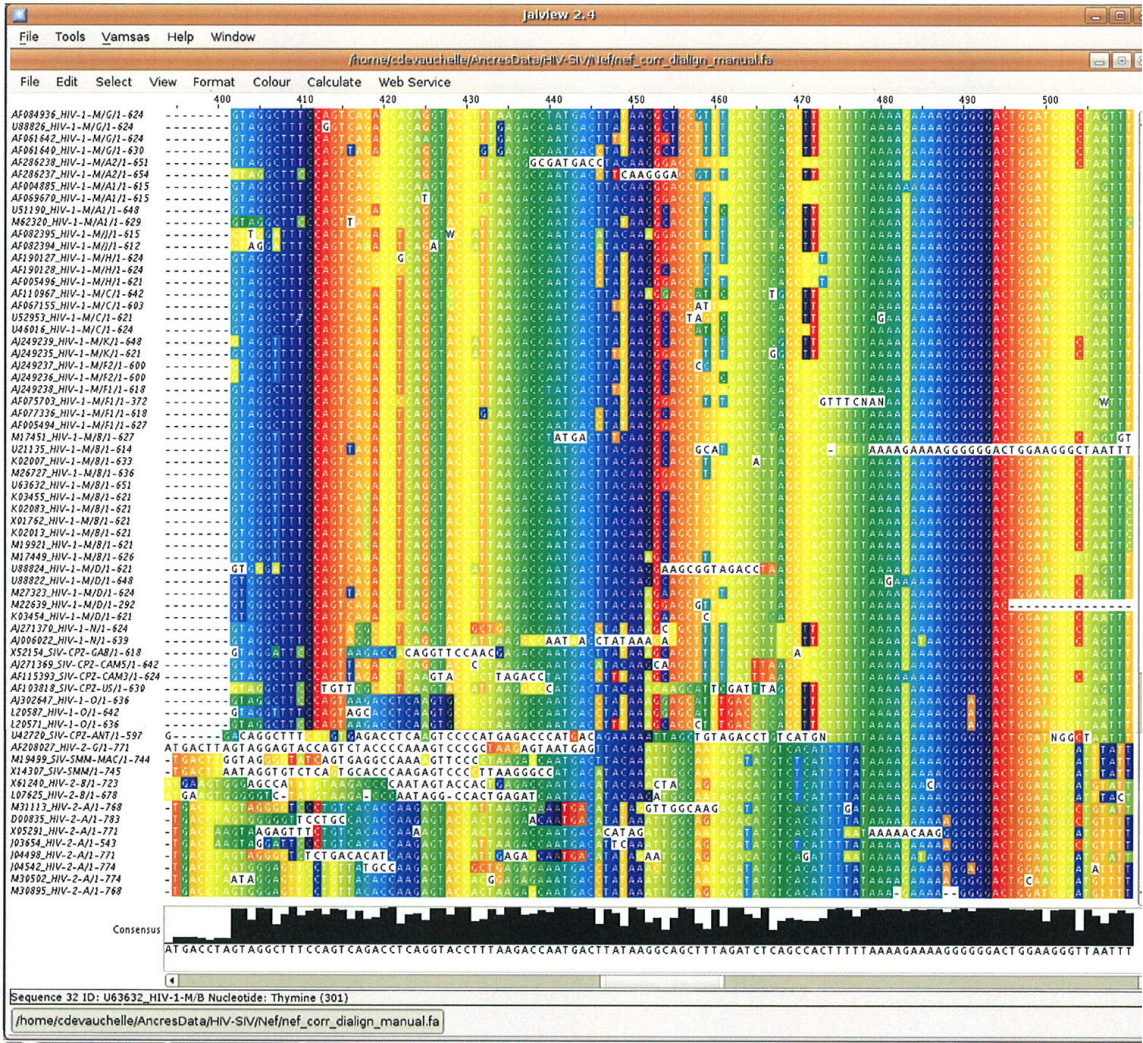
**Screenshot of local *nef *alignment**. Jalview screenshot of positions 402 to 510 of the alignment of 66 *nef *sequences. Identical letters (A, C, G or T) that are of the same color and on the same column, come from the same MS4 class. (It can happen that two neighboring colors are hard to distinguish). In the left column, the sequences are identified by their accession number, the type of virus (HIV1 or 2, SIV-CPZ or SMM) the group -for example HIV-1 M, N, O or HIV-2-A, the subtype, and the subtype in the case of HIV-1-M/C, for example. The sites that are not colored belong to classes with only one element.

Here we examine more precisely *nef*, a sequence which is important for the virulence of the virus. We show a multiple alignment of the 66 sequences (Fig. 7). The Dialign [19] multiple alignment has been manually edited by putting in the same column the sites corresponding to one MS4 class (See section Methods). The results have been visualized with the help of Jalview [20] which is a multiple alignment editor which allows the user to define, for each color, the set of sites that carry that color. The fig. 7 shows an unambiguous sector of this alignment. The identifiers of the classes are not shown on the figure, but Jalview fortunately allows the user to click on a letter and recover this information. Identical letters (A, C, G or T) that are on the same column and with the same colour belong to the same class. We clearly see on Fig. 7 that there are classes that appear only in HIV-1, classes that appear only in HIV2, and classes that appear in both. The fact that sequences can be correctly classified by MS4, suggests that the majority of sites regrouped in one class correspond to blocks of homology between sequences.

## Conclusions

This paper gives a description of the MultiScale Selector of Sequence Signatures (MS4) method and uses it for an alignment-free classification (virtually parameter-free) of a family of sequences. The core of the method consists in the selection of "relevant" classes of segments, which are assumed to carry similarity information, although the criterion for grouping them together is purely combinatorial (classification by context [9]). The point of our method is that it does not require the specification of a word length parameter and it does not consider only exact words.

The user may choose a parameter *κ *which reflects the average repetitivity of the set of sequences under consideration. The default value *κ *= 1 yields satisfying results in the examples we have considered so far. MS4 sets automatically a local length parameter *N *which depends on the starting set of sequences and local similarities between sequences.

In this paper, we test the method on a set of well-studied HIV/SIV sequences [10,14,16] on which one of us is an expert [10,18]. The results obtained are in excellent agreement with the accepted knowledge. The MS4 method has also been applied to other data (not shown here). It should be noted that it is not accurate on too *small *datasets. In our experience, this program can be applied in its present state to sets composed from a dozen to a few hundreds of sequences (datasets consisting of a few Mb). Note also that MS4 works for protein data as well as genes (e.g. Additional File 11, and [21]).

As *N *decreases, the *N*-local decoding method detects weaker similarities, before being flooded by spurious ones [13]. Concerning the selection of equivalence classes, our aim is to select as many non-redundant homologous segments as possible, while keeping the background noise at a low level. Our default criterion for "relevant" classes locally sets *N *above this level, at the cost of losing some occurrences of repeated similar segments. By tuning the parameter *κ*, it is possible to accept a maximal average quantity of repetitions below a given threshold. When *κ *is set too high, the result of the classification can degenerate, and tends towards the mere letter-composition criterion as *κ *tends to infinity. By default, we exclude repetitions of any given class in the same sequence. However, even for this value, the repeated segments are not lost altogether. When the value of *N *becomes larger than the size of the repetition, the MS4 classes only change (as subsets of sites) up to the value where different repetitions are assigned a different MS4 class. This can indeed result in a clearer identification of the distinct homologous repetitions. This phenomenon is illustrated on the well known repetitive NF*κ*B binding regions of non-coding LTR (see Fig. 4 and Section Results sub-section NF*κ*B region). Although our current criterion can be tuned to take repetitivity into account, the classifications of the HIV/SIV sequences turn out to be remarkably robust to the variations of the parameter *κ *(for example see in additional files 6 and 7 the resulting SplitsTree from non coding part of LTR sequences for *κ *= 5 and 10). Nevertheless, it seems desirable to get a more significant criterion, statistical-based, to prune the tree formed by the whole set of embedded partitions (See section Methods subsection Partition Tree and Choice of classes). The last step concerns the computation of the similarity matrix. Our similarity is straightforward: it consists in counting the number of MS4 classes that are shared by 2 sequences. This corresponds to a usual basic scheme for the comparison of two nucleic sequences (% identity). We group together similar sites (according to MS4) as equivalence classes. As a result, a segment of identity of length *N *between sequences will result in *N *MS4 classes (Additional Files 8 and 9). Each MS4 class has an equal weight in our dissimilarity computation (See Eq.3). In the case of an exact repeated subword of length *N *between two sequences, the contribution of this subword to the dissimilarity is exactly *N*.

However, it could be also possible in the future to obtain a SplitsTree by constructing directly the splits themselves on the basis of the selected segment classes, and to avoid the computation of the matrix. The presence of incompatible signals (resulting in parallelograms) in the network constructed by SplitsTree4 [11] from MS4 similarity matrices for short sequences, shows, as otherwise expected, that this method must usually be completed by visual expertise. This can be achieved by coupling MS4 with multiple alignment editor like Jalview [22] (See Fig. 6 and Fig. 7). Therefore, the classes detected by MS4 can be used to help the manual editing of a multiple alignment. We also use them to determine anchor points for the multiple alignment programs [21].

## Availability

A user-friendly Web-interface is available at http://stat.genopole.cnrs.fr/ms4/. It takes as input a file with sequences in fasta format and gives the dissimilarity matrix in nexus format to run the option NeighborNet of SplitsTree4. The allowed parameters are *κ *(default value 1) and the range of N for computing the partition tree (default values: from 2 to *N*_max _which is the size of the maximal repeated word shared by two sequences in the dataset). The Python code is avalaible in Additional File 12 and upon request from the corresponding author (for some implementation details see the algorithm in section Appendix).

## Authors' contributions

EC and FP conceived the method and wrote part of the code, GG made the code available and implemented the Web interface, IL gave the original idea for the biological application and expertised the results, GD wrote part of the code, CD and EC drafted the manuscript, CD produced the results, expertised them, and supervised this work. All authors read and approved the final manuscript.

## Supplementary Material

Additional file 1 **Network for Compendium2000 sequences**. Network for the 46 Compendium2000 sequences computed by SplitsTree4 on our MS4 dissimilarity matrix with *κ *= 1 (from *N *= 2 to *N *= 60). Click here for file

Additional file 2 **Network for *gag *sequences**. Network for the 70 *gag *sequences computed by SplitsTree4 on MS4 dissimilarity matrix with *κ *= 1 (*N*_max _= 510). Click here for file

Additional file 3 **Network for the *pol *sequences**. Network for the 66 *pol *sequences computed by SplitsTree4 on MS4 dissimilarity matrix with *κ *= 1 (*N*_max _= 962). Click here for file

Additional file 4 **Network for *env *sequences**. Network for the 66 *env *sequences computed by SplitsTree4 on MS4 dissimilarity matrix with *κ *= 1 (N_max _= 794). Click here for file

Additional file 5 **Network for LTR sequences obtained with NLD**. The SplitsTree4 network for non-coding LTR sequences computed with the NLD method for a fixed word length of *N *= 11. NLD method is described in [10], it uses a similar similarity index but with a fixed length word. In [10] we used Neighbor Joining instead of Splits Networks. Click here for file

Additional file 6 **SplitsTree network for *k *= 5 for LTR sequences**. Network for the 43 non coding sequences parts of HIV LTR computed by SplitsTree4 on MS4 dissimilarity matrix for the value *κ *= 5 (*N *from 2 to 100). Click here for file

Additional file 7 **SplitsTree network for *k *= 10 for LTR sequences**. Network for the 43 non coding sequences parts of HIV LTR computed by SplitsTree4 on MS4 dissimilarity matrix for the value *κ *= 10 (*N *from 2 to 100). Click here for file

Additional file 8 **Similarity blocks found by MS4 in non coding LTR sequences**. Superposition of MS4 classes on a manually expertised alignment of the non coding part of 43 HIV-SIV LTR sequences focused on NF*κ*B region. This is a nucleotide sequences alignment of the 43 non-coding LTR sequences. Apart from minor modifications the alignment is the same as that in Fig. 5 in [10]. The alignment is focused on the transcription factor NF*κ*B binding site (GGGACTTTCC[A|G]) and its flanking regions. The names of sequences are indicated with their accession number in Los Alamos HIV sequence databank. The sequence are regrouped according to their phylogeny. The letters are rewritten by applying the MS4 method to the whole non coding LTR sequences. The MS4 identifier is constructed as follows: e.g. C24_8 (class C24 for a N value of 8). Identical recoded letters that are in the same columns are displayed in the same colour. When they are not all aligned on the same column no colour is used (as well as when they are unique in this part of the alignment). The repeated motifs inside one sequence are put one under the other. Therefore the sequences are often written on several lines to highlight similarities between sequences and inside sequences. Most often the similarity blocks are aligned and the great majority of identical indexed letters are on only one column. Click here for file

Additional file 9 **Region of NF*κ *B fixation site**. The complete alignment, part of which is featured in Fig. 4. This figure corresponds to the figure in Additional File 8. The colours are the same as in the figure in Additional File 8 but in this figure the MS4 identifier has been simplified as follows: we have just indicated the letter and the value of *N*. Therefore it can be that two different MS4 classes that lie on the same column, with the same letter and the same *N *value are only distinguished by their colour (e.g. A18 and also T18 HIV-1-M/G, that are red or green). Click here for file

Additional file 10 **Network for the *nef *sequences**. Network for the 66 *nef *nucleic sequences computed by SplitsTree4 on MS4 dissimilarity matrix with *κ *= 1 (for *N*_max _= 543). Click here for file

Additional file 11 **Network for the Nef protein sequences**. Network for the 66 Nef protein sequences on MS4 dissimilarity matrix with *κ *= 1 (for *N *= 2 to *N *= 100). Click here for file

Additional file 12 **Python code source**. Python implementation of MS4 algorithm for linux systems. See INSTALL and README files to use it. Click here for file

## Appendix

**Algorithm 1 **Main steps to select relevant classes in the partition tree

Input: All equivalence classes *E *∈ ℰ*^n^*, for *n *∈ {*n_min_*, ..., *n_max_*}

Input: ∑ set of all sites

1:

2: // Initialize the MS4 equivalence classes

3: RelevantECList←{∅}

4:

5: // Initialize the partition tree *P*: add leaves (∈ℰ^∞^) in *P*

6: **for each **site *σ *∈ ∑, ∑ set of sites **do**

7:    // Add a new node in the partition tree *P *and initialize *κ*

8:    addNode(*P*, *σ*)

9:    *κ *(σ)←1

10: end

11:

12: // Main loop

13: n ← *n*_max_

14: **while ***n *≥ *n_min _***do**

15:    **for each **equivalence class *E *∈ ℰ*^n^***do**

16:       // Build *A*, the highest ancestor set of *E *in *P*

17:        ← {∅}

18:       **for each **site *σ *∈ *E ***do**

19:           ← ∪ getHighestAncestor(*P, σ*)

20:       **end**

21:       // Compact the partition tree if only one ancestor is found

22:       **if ***card*() > 1 **then**

23:          // Add a new node in the partition tree *P*

24:          addNode (*P*, *E*)

25:          // Compute *κ*(*E*): (*E*) is the number of sequences where equivalence class *E *appears

26:          *κ *(*E*) ← *card*(*E*)/(*E*)

27:          **for each **equivalence class A ∈ **do**

28:             // Set inclusion relation *A *⊂ *E *in the partition tree *P*

29:             addEdge(*P*, (*E, A*)).

30:             **if ***κ *(E) <*κ *and *κ*(A) ≥ *κ ***then**

31:                RelevantECList ← RelevantECList ∪{*A*}

32:             **end**

33:          **end**

34:       **end**

35:    **end**

36:    *n *← *n *- 1

37: **end while**

38:

39: // Create *root *node (*i.e. *ℰ^0^), connect it to the highest ancestors in *P*

40: // Same as above

41:

42: **return **RelevantECList
