# Supplementary figures and images for: MS4 - Multi-Scale Selector of Sequence Signatures: An alignment-free method for classification of biological sequences
Source: BMC Bioinformatics. 2010 Jul 30;11:406. doi: 10.1186/1471-2105-11-406 (PMC2923138; doi:10.1186/1471-2105-11-406)

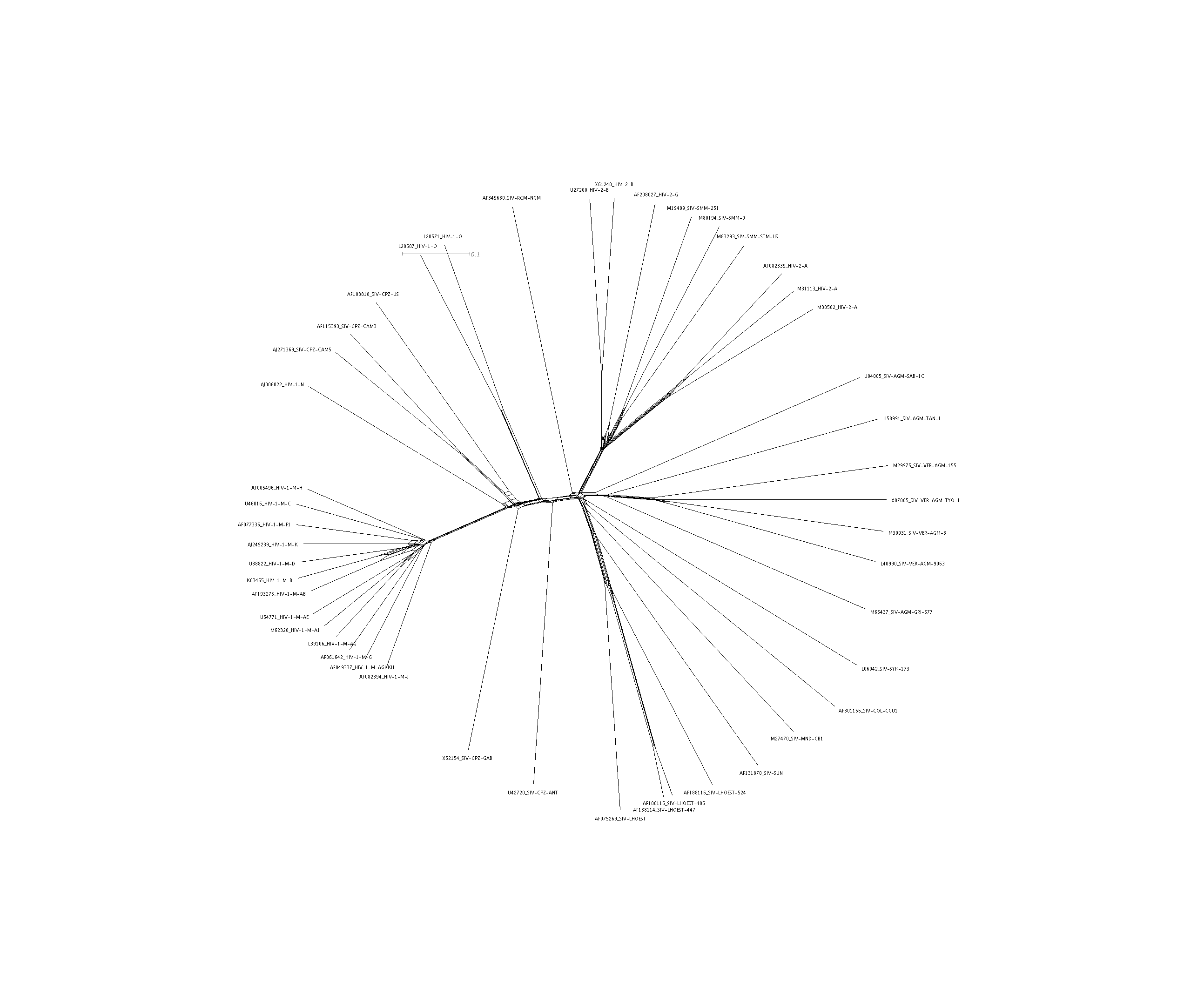

Supplement: Additional file 1 — Network for Compendium2000 sequences. Network for the 46 Compendium2000 sequences computed by SplitsTree4 on our MS4 dissimilarity matrix with κ = 1 (from N = 2 to N = 60). [file 1471-2105-11-406-S1.PNG]

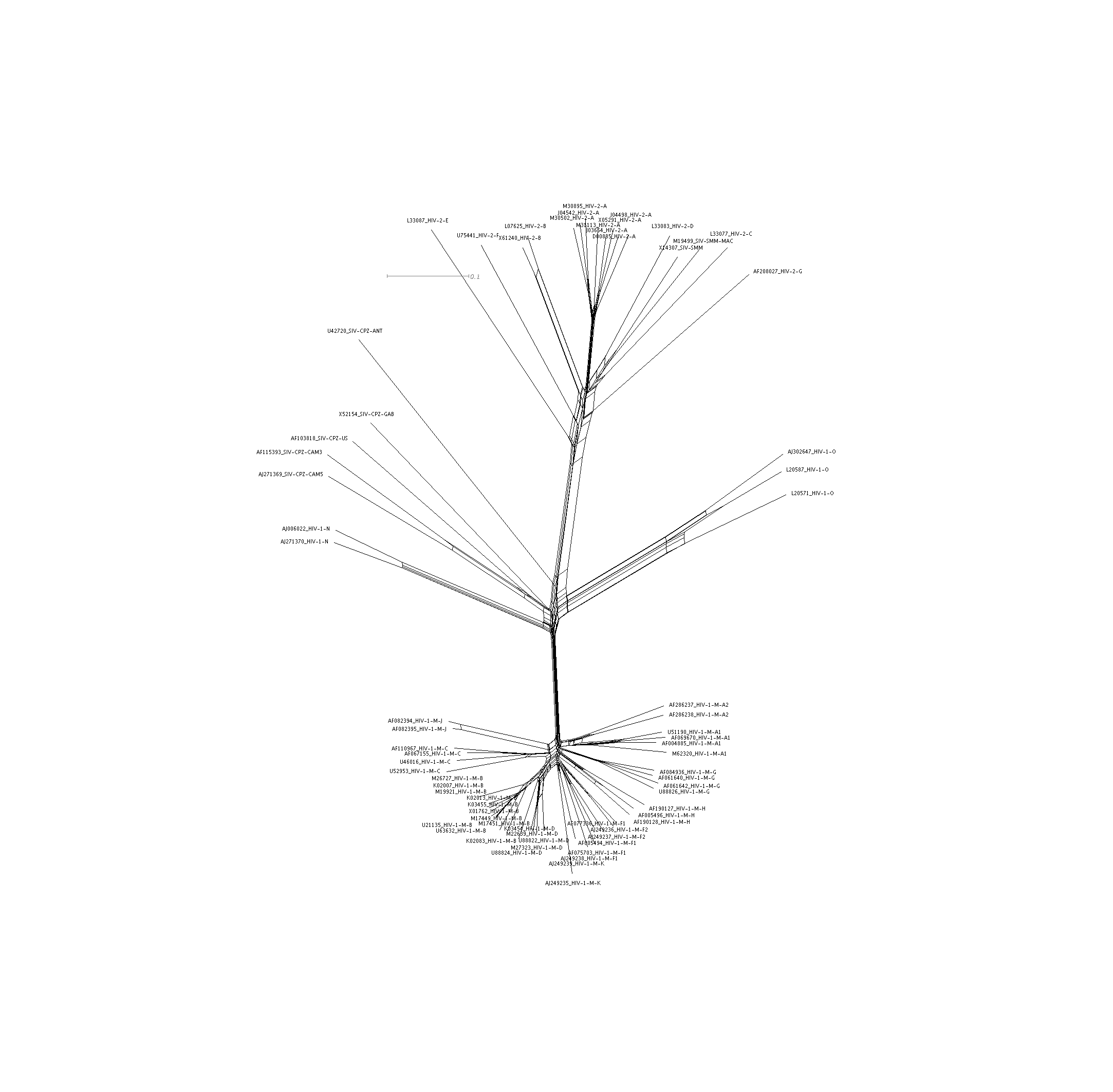

Supplement: Additional file 2 — Network for gag sequences. Network for the 70 gag sequences computed by SplitsTree4 on MS4 dissimilarity matrix with κ = 1 (Nmax = 510). [file 1471-2105-11-406-S2.PNG]

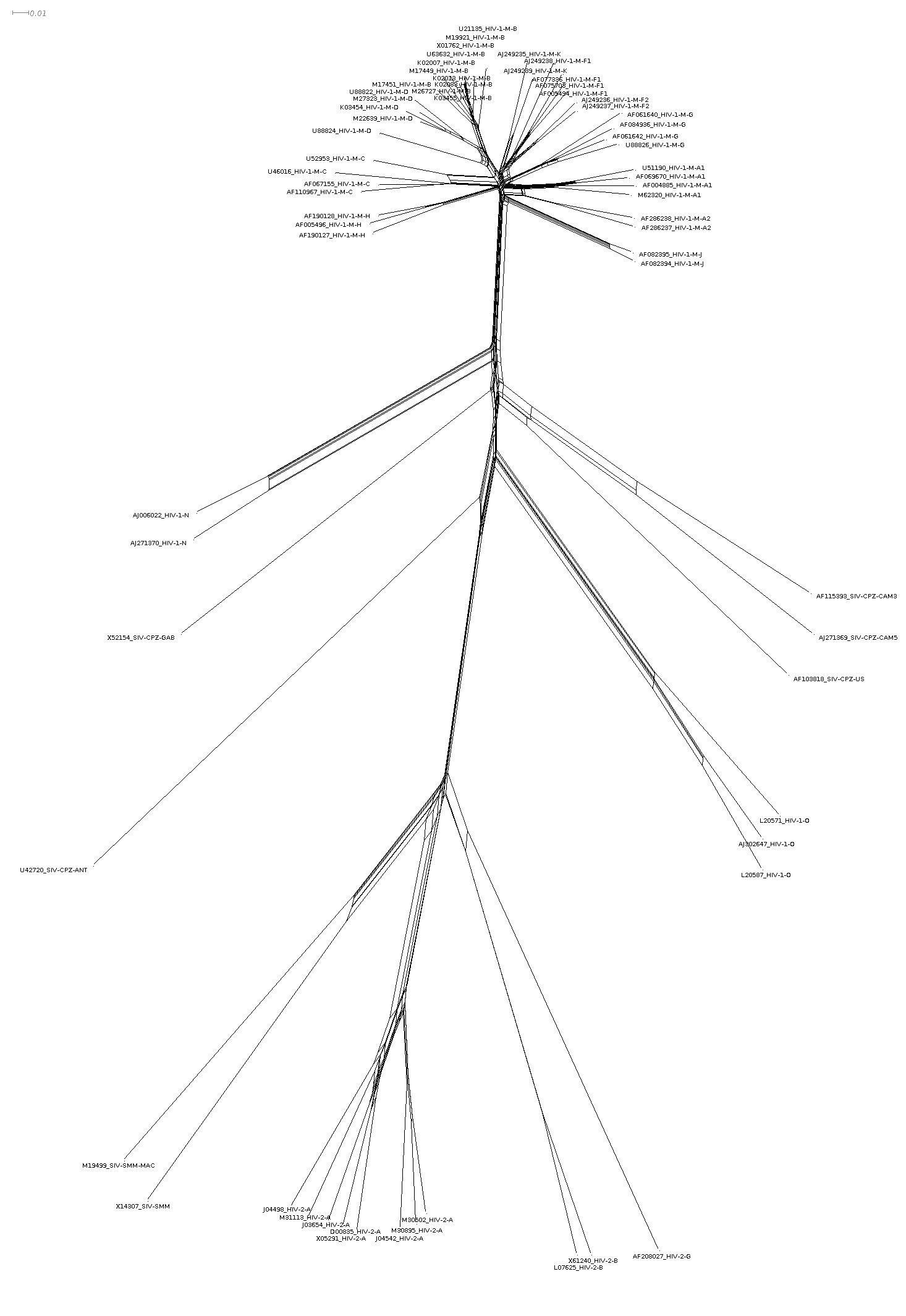

Supplement: Additional file 3 — Network for the pol sequences. Network for the 66 pol sequences computed by SplitsTree4 on MS4 dissimilarity matrix with κ = 1 (Nmax = 962). [file 1471-2105-11-406-S3.PNG]

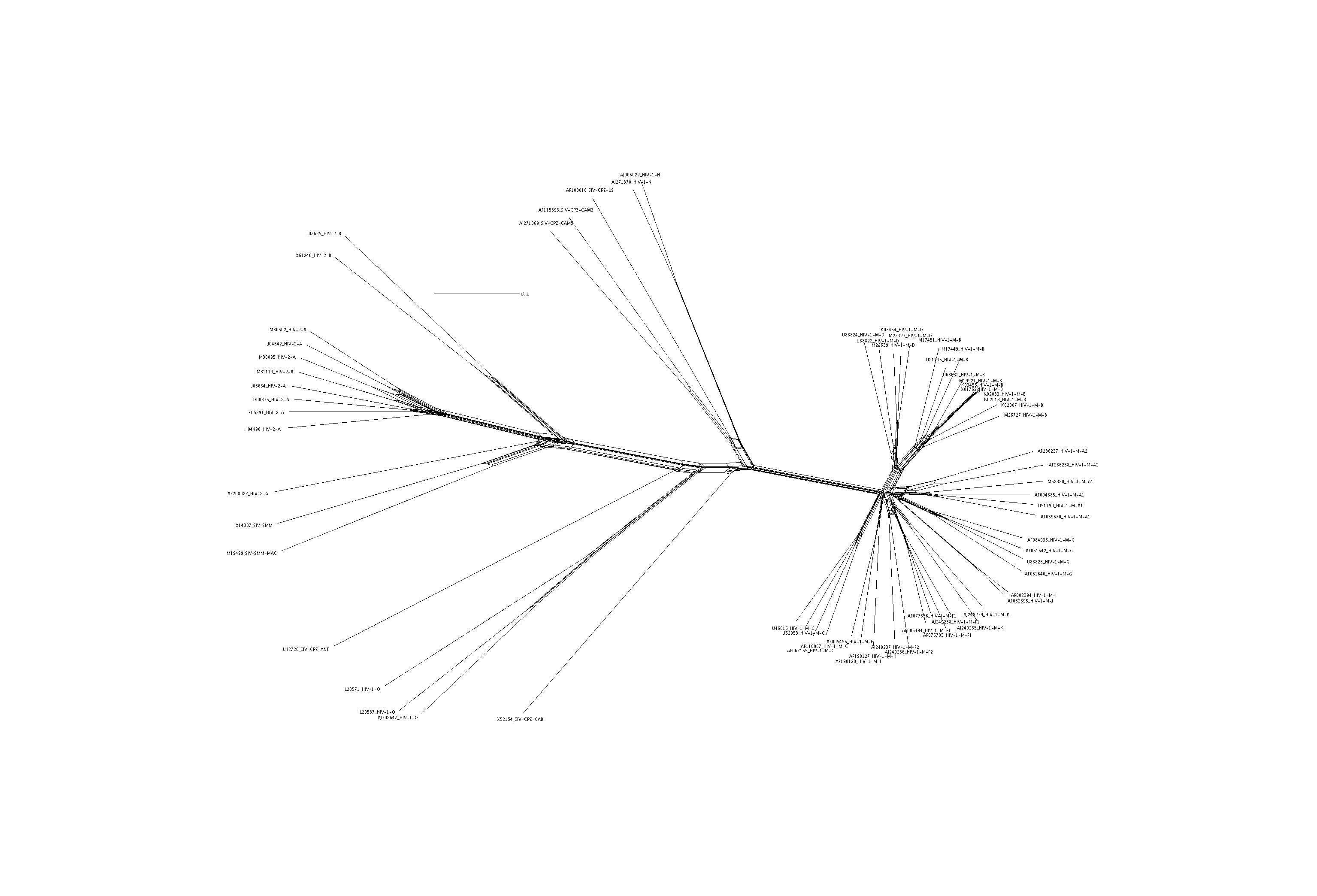

Supplement: Additional file 4 — Network for env sequences. Network for the 66 env sequences computed by SplitsTree4 on MS4 dissimilarity matrix with κ = 1 (Nmax = 794). [file 1471-2105-11-406-S4.PNG]

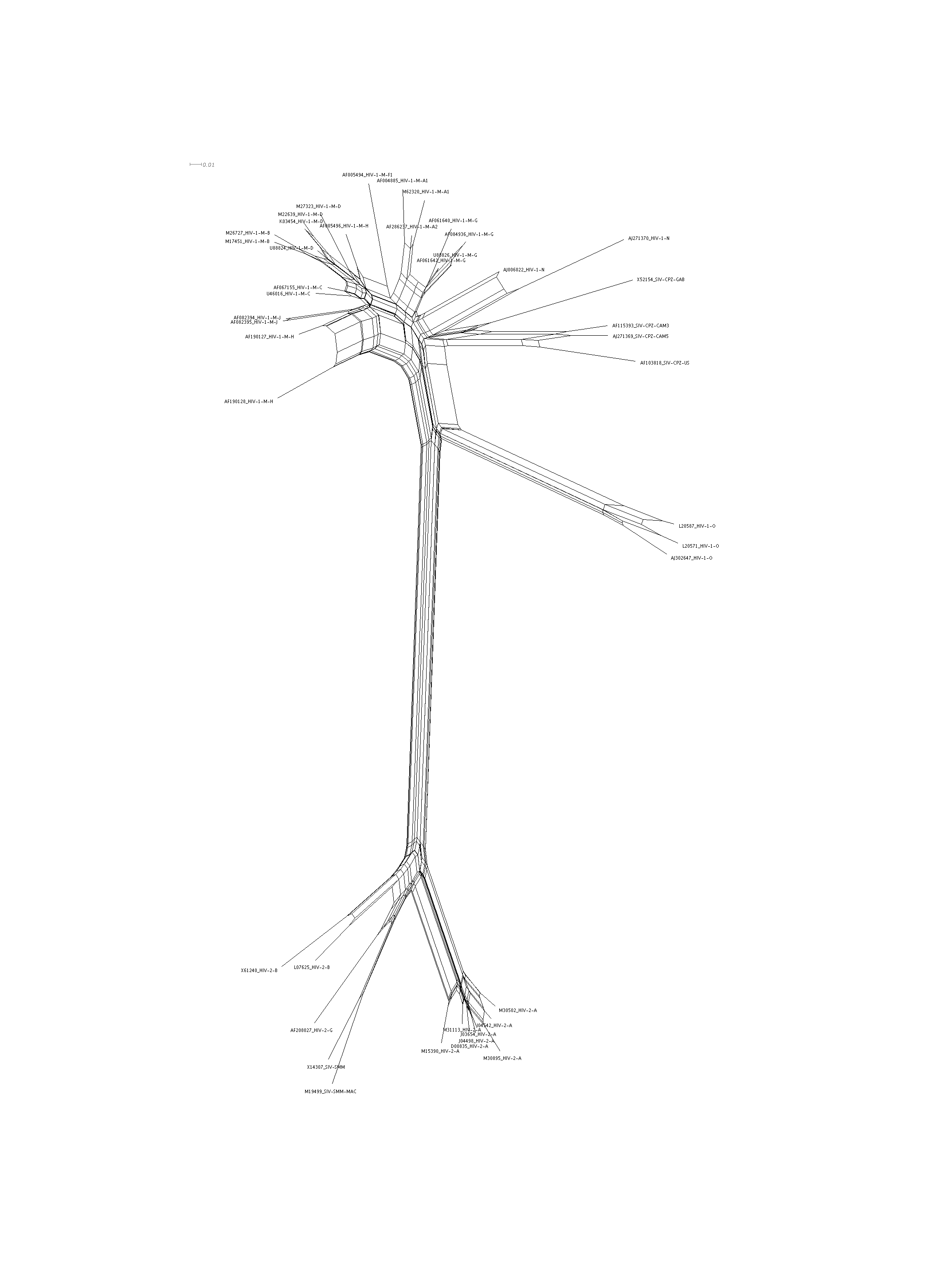

Supplement: Additional file 5 — Network for LTR sequences obtained with NLD. The SplitsTree4 network for non-coding LTR sequences computed with the NLD method for a fixed word length of N = 11. NLD method is described in [10], it uses a similar similarity index but with a fixed length word. In [10] we used Neighbor Joining instead of Splits Networks. [file 1471-2105-11-406-S5.PNG]

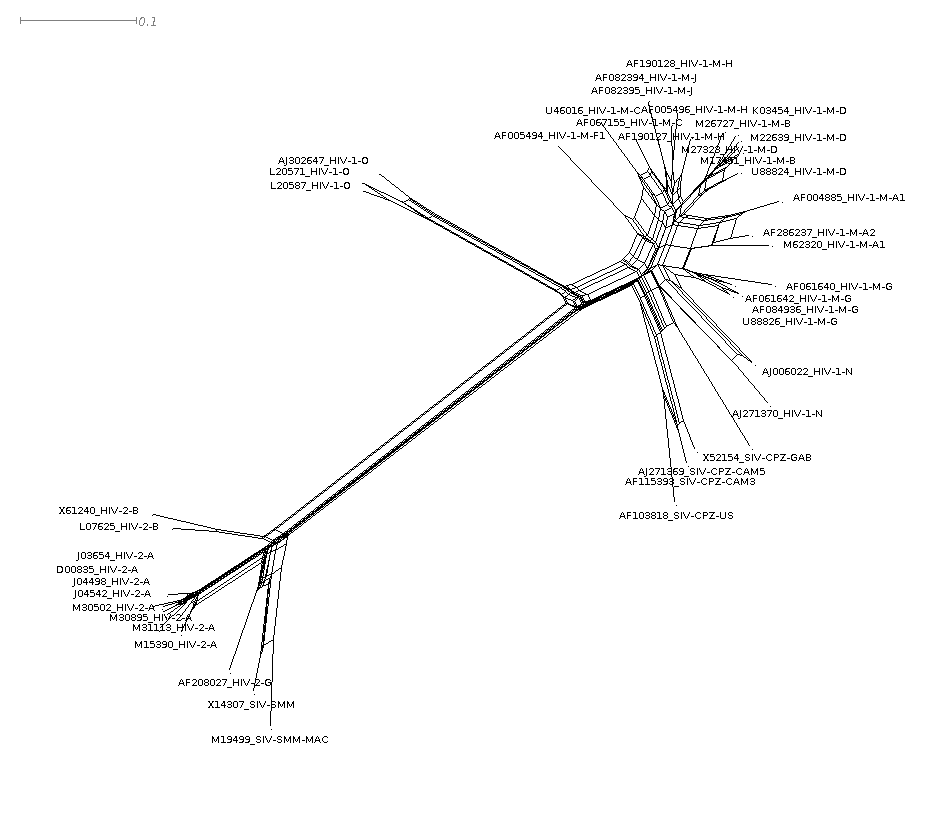

Supplement: Additional file 6 — SplitsTree network for k = 5 for LTR sequences. Network for the 43 non coding sequences parts of HIV LTR computed by SplitsTree4 on MS4 dissimilarity matrix for the value κ = 5 (N from 2 to 100). [file 1471-2105-11-406-S6.PNG]

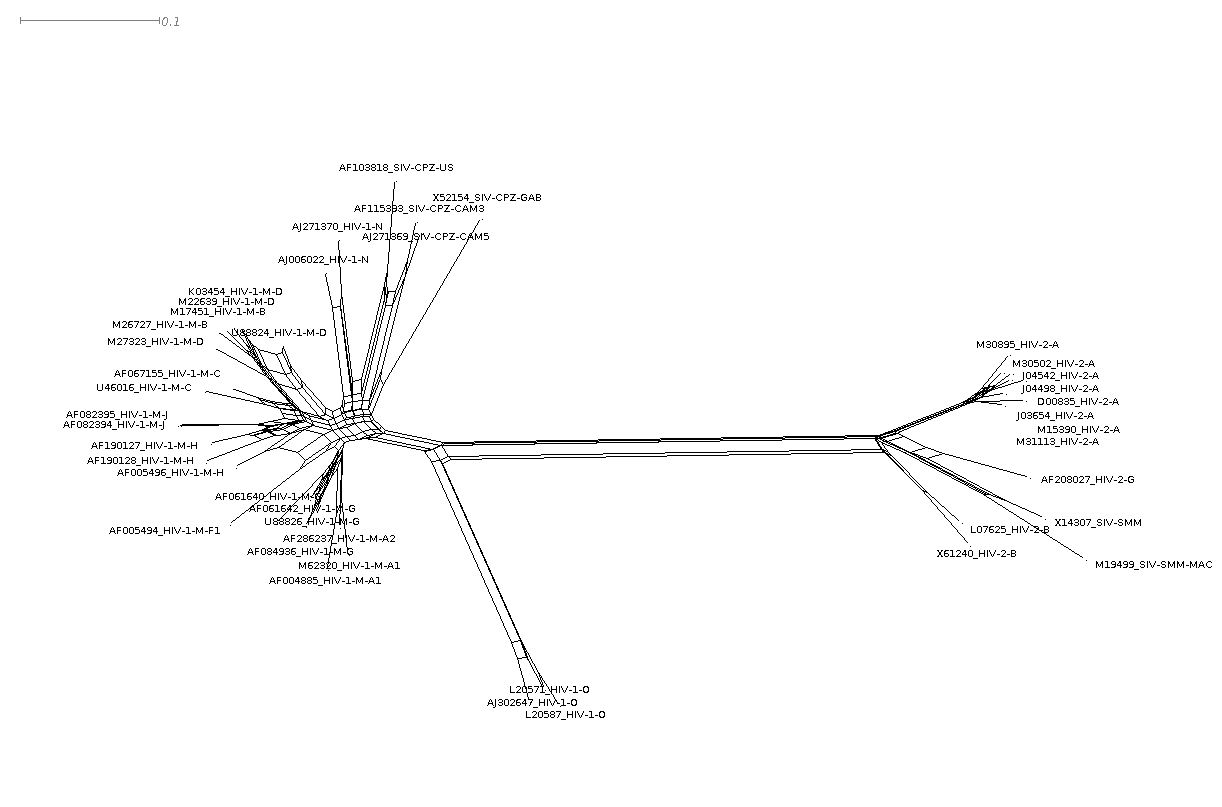

Supplement: Additional file 7 — SplitsTree network for k = 10 for LTR sequences. Network for the 43 non coding sequences parts of HIV LTR computed by SplitsTree4 on MS4 dissimilarity matrix for the value κ = 10 (N from 2 to 100). [file 1471-2105-11-406-S7.PNG]

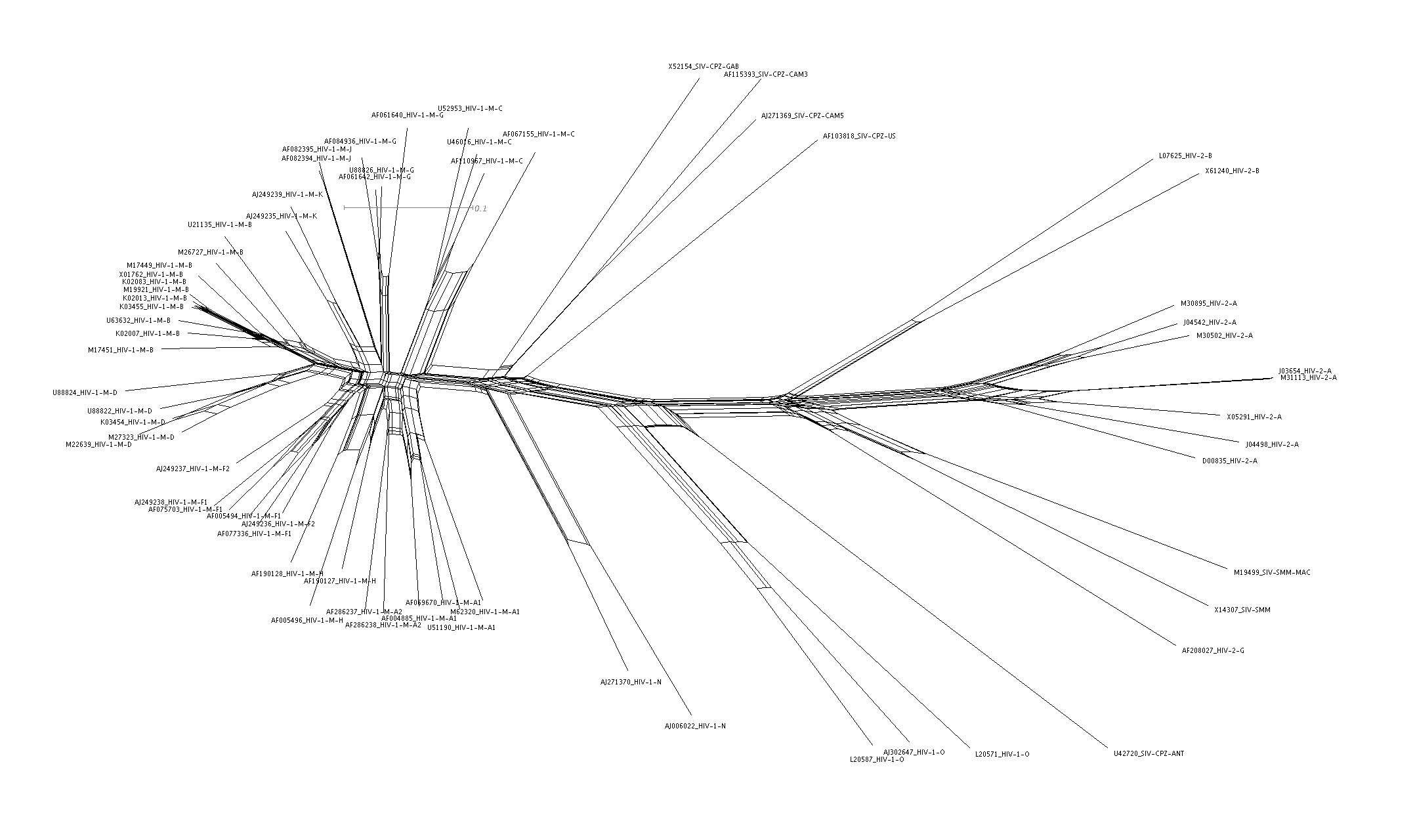

Supplement: Additional file 10 — Network for the nef sequences. Network for the 66 nef nucleic sequences computed by SplitsTree4 on MS4 dissimilarity matrix with κ = 1 (for Nmax = 543). [file 1471-2105-11-406-S10.PNG]

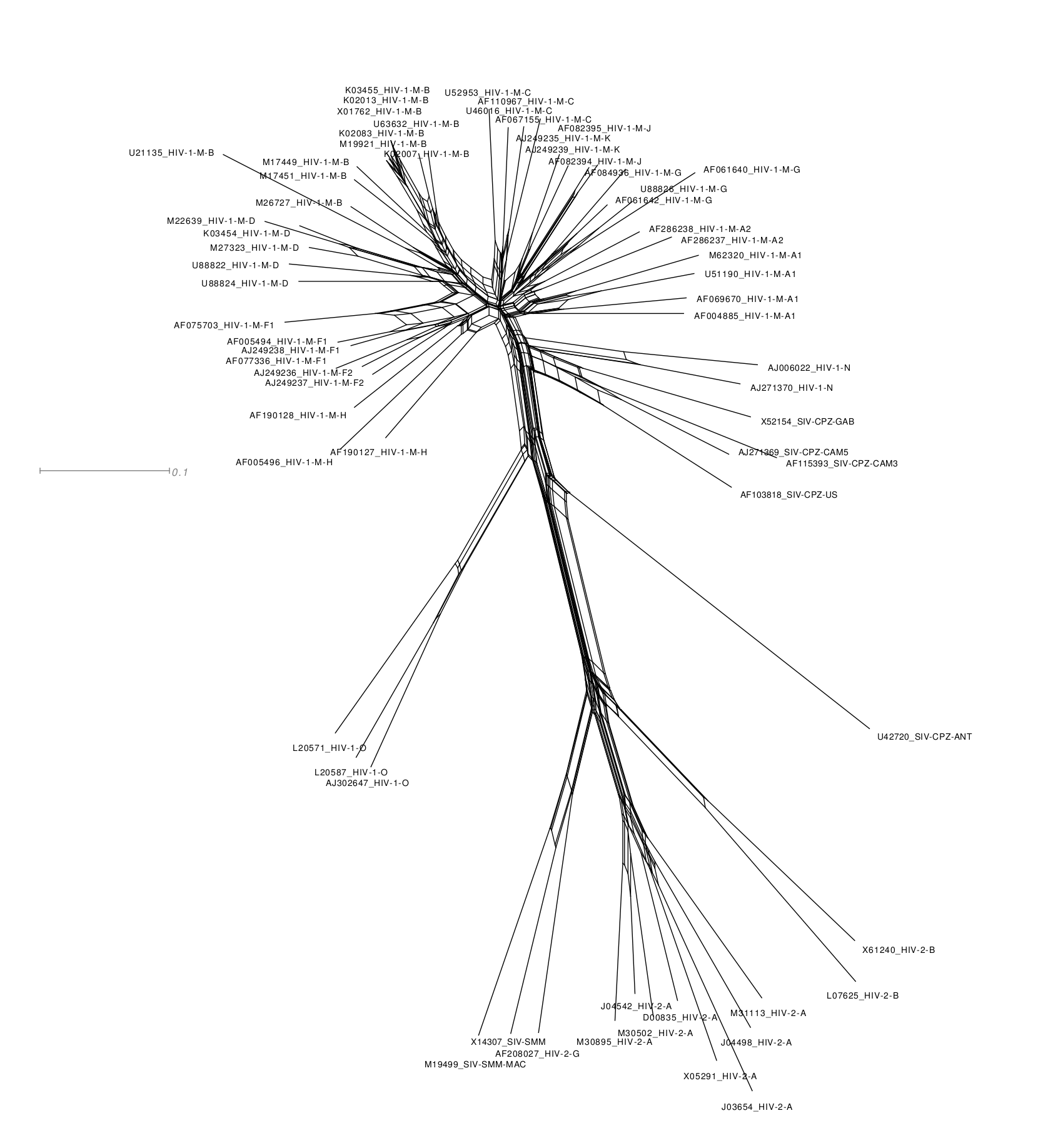

Supplement: Additional file 11 — Network for the Nef protein sequences. Network for the 66 Nef protein sequences on MS4 dissimilarity matrix with κ = 1 (for N = 2 to N = 100). [file 1471-2105-11-406-S11.PNG]
