# Supplementary material for: MS4 - Multi-Scale Selector of Sequence Signatures: An alignment-free method for classification of biological sequences
Source: BMC Bioinformatics. 2010 Jul 30;11:406. doi: 10.1186/1471-2105-11-406 (PMC2923138; doi:10.1186/1471-2105-11-406)
Supplement: Additional file 9 — Region of NFκ B fixation site. The complete alignment, part of which is featured in Fig. 4. This figure corresponds to the figure in Additional File 8. The colours are the same as in the figure in Additional File 8 but in this figure the MS4 identifier has been simplified as follows: we have just indicated the letter and the value of N. Therefore it can be that two different MS4 classes that lie on the same column, with the same letter and the same N value are only distinguished by their colour (e.g. A18 and also T18 HIV-1-M/G, that are red or green). [file 1471-2105-11-406-S9.PDF]

>AF067155_HIV-1-M/C 1 C A C G G A18 A18 G18 G18 G18 A18 C18 T18 T18 T18 C18 C18 G15
C16 T18 G21 G21 G21 A18 C18 T18 T18 T18 C18 C18 A16
C16 T16 G16 G16 G16 C15 G15 T15 T15 C15 C15 A15 G15 G15 A13 G13 G13 T10 G10

>U46016_HIV-1-M/C 1 C A C A G A C G G G A C T T T C C G
C C G17 G17 G17 A17 C17 T17 T17 T17 C17 C17 A16
C16 T16 G16 G16 G16 G17 C15 G15 T15 T15 C15 C15 A15 G15 G15 A13 G13 G13 A G

>M27323_HIV-1-M/D 4 C10 G10 A10 G10 T10 T10 T10 C C13 T13 A11 C15 A18 A18 G18 G18 G18 A18 C18 T18 T18 T18 C18 C18 G15
C16 T18 G30 G21 G21 G21 A18 C18 T18 T18 T18 C18 C18 A15 G23 G23 G23 A13 G13 G13

>U88824_HIV-1-M/D 4 C8 G13 A13 G13 C13 T10 T10 T13 C13 T13 A11 C15 A18 A18 G18 G18 G18 A18 C18 T18 T18 T18 C18 C18 G15
C16 T18 G30 G21 G21 G21 A18 C18 T18 T18 T18 C18 C18 A15 G23 G23 G23 A13 G13 G13

>K03454_HIV-1-M/D 7 C8 G13 A13 G13 C13 T10 T10 T13 C13 T13 A11 C15 A18 A18 G18 G18 G18 A18 C18 T18 T18 T18 C18 C18 G15
C16 T18 G30 G21 G21 G21 A18 C18 T18 T18 T18 C18 C18 A15 G23 G23 G23 A13 G13 G13

>M22639_HIV-1-M/D 4 C A A10 G10 T10 T10 T10 T13 C13 T13 A11 C15 A18 A18 G18 G18 G18 A18 C18 T18 T18 T18 C18 C18 G15
C16 T18 G30 G21 G21 G21 A18 C18 T18 T18 T18 C18 C18 G21 G23 G23 G23 A13 G13 G13

>M17451_HIV-1-M/B 4 C10 G10 A10 G10 T10 T10 T10 T13 C13 T13 A11 C15 A18 A18 G18 G18 G18 A18 C18 T18 T18 T18 C18 C18 G15
C16 T18 G30 G21 G21 G21 A18 C18 T18 T18 T18 C18 C18 A15 G23 G23 G23 A13 G13 G13

>M26727_HIV-1-M/B 4 C8 G13 A13 G13 C13 T10 T10 T13 C13 T13 A11 C A G15 G18 G18 G18 A18 C18 T18 T18 T18 C18 C18 G15
C16 T18 G30 G21 G21 G21 A18 C18 T18 T18 T18 C18 C18 A15 G23 G23 G23 A13 G13 G13

>AF082395_HIV-1-M/J 4 A49 G49 A49 A49 G49 T49 T49 T49 C49 T49 A49 G49 C15 G15 G18 G18 G18 A18 C18 T18 T18 T18 C18 C18 G15
C16 T18 G30 G21 G21 G21 A18 C18 T18 T18 T18 C18 C18 A15 G23 G23 G23 G34 A13 G13

>AF082394_HIV-1-M/J 4 A49 G49 A49 A49 G49 T49 T49 T49 C49 T49 A49 G49 C15 G15 G18 G18 G18 A18 C18 T18 T18 T18 C18 C18 G15
C16 T18 G30 G21 G21 G21 A18 C18 T18 T18 T18 C18 C18 A15 G23 G23 G23 G34 A13 G13

>AF190128_HIV-1-M/H 4 A10 G10 A10 A9 G9 A9 T10 T12 C11 T11 G11 A11 C15 A18 G18 G18 G18 A18 C18 T18 T18 T18 C18 C18 G15
C16 T18 G21 G21 G21 A18 C18 T18 T18 T18 C18 C18 G21 G24 G24 G24 A13 G13 G13

>AF190127_HIV-1-M/H 5 G10 A10 A9 G9 A9 T10 T12 C11 T11 A T A11 C15 A18 G18 G18 G18 A18 C18 T18 T18 T18 C18 C18 G15
C16 T18 G21 G21 G21 A18 C18 T18 T18 T18 C18 T10 G G G15 G15 A13 G13 G13

>AF005496_HIV-1-M/H 3 C10 A10 G10 A10 A9 G9 A9 T10 C T G A13 A18 A18 G18 G18 G18 A18 C18 T18 T18 T18 C18 C18 G15
C16 T18 G21 G21 G21 A18 C18 T18 T18 T18 C18 C18 G21 G24 G24 G24 A13 G13 G13

>AF005494_HIV-1-M/F1 27 C A G A A9 G9 A9 A8 T8 C T A13 A A17 G17 G17 G17 A17 C17 T17 T17 T17 C17 C17 A16
C16 T16 G16 G16 G16 G17 A25 C25 T25 T25 T25 C25 C25 A15 G A G G G T7

>AF061642_HIV-1-M/G 5 G15 A15 A9 G9 T9 T11 G11 C11 T11 G11 A11 C15 A17 A18 G26 G26 G26 G26 A18 C18 T18 T18 T18 C18 C27 A
C C16 T18 G21 G21 G21 A18 C18 T18 T18 T18 C18 C18 G21 G24 G24 G24 A13 G13 G13

>AF061640_HIV-1-M/G 5 G15 A15 A9 G9 T9 T11 G11 C11 T11 G11 A11 C15 A17 A18 G26 G26 G26 G26 A18 C18 T18 T18 C C C G15
C16 C16 T16 G G G A C T T T C C G G A10 G A13 G13 G13

>AF084936_HIV-1-M/G 5 G15 A15 A9 G9 T9 T11 G11 C11 T11 G11 A11 C15 A17 A18 G26 G26 G26 G26 A18 C18 T18 T18 T18 C18 C27 G15
C16 C16 T16 G27 G27 G27 A18 C18 T18 T18 T18 C18 C18 A15 G21 G27 G G91 A13 G13

>U88826_HIV-1-M/G 5 G15 A15 A9 G9 T9 T11 G11 C11 T11 G11 A11 C15 A17 A18 G26 G26 G26 A18 C18 T18 T18 T18 C18 C27 G15
C16 C16 T16 G27 G27 G27 A18 C18 T18 T18 T18 C18 C18 A15 G21 G27 A10 G91 A13 G13

>AF286237_HIV-1-M/A2 4 A15 G15 A15 A9 G9 T9 T11 G11 C11 T11 G11 A11 C15 G15 G18 G18 G18 A18 C18 T18 T18 T18 C18 C18 G15
C16 T18 G30 G21 G21 G21 A18 C18 T18 T18 T18 C18 C18 A15 G23 G23 G23 A13 G13 G13

>AF004885_HIV-1-M/A1 4 A G A A9 G9 T9 T11 G11 T7 T7 G A C T G18 G18 G18 A18 C18 T18 T18 T18 C18 C18 G15
C16 T18 G30 G21 G21 G21 A18 C18 T18 T18 T18 C18 C18 A15 G23 G23 G23 G34 A13 G13

>M62320_HIV-1-M/A1 15 A15 G15 A15 A9 G9 T9 T11 G11 C11 T11 G11 A11 C15 A17 G17 G17 G17 A17 C17 T17 T17 T17 C17 C17 A16
C16 T16 G16 G16 G16 G17 A25 C25 T25 T25 T25 C25 C25 G A G23 G23 G34 A13 G13

>AJ271369_SIV-CPZ-CAM5 1 A11 A11 A13 C11 T11 G11 C11 T11 G11 A11 C15 A17 A18 G19 G19 G19 A18 C18 T18 T18 T18 C18 T10 A26
A26 G17 G17 G17 A15 C15 T15 T15 T15 C15 C15 A15
A17 G17 G17 G17 G11 G11 A G G C T9 C12 A12 G12 G12 G12 C12 G12

>AF115393_SIV-CPZ-CAM3 1 A11 A11 A13 C11 T11 G11 C11 T11 G11 A11 C15 A17 A18 G19 G19 G19 A18 C18 T18 T18 T18 C18 T10 A26
A26 G17 G17 G17 A15 C15 T15 T15 T15 C15 C15 A15
A17 G17 G17 G17 G11 G11 G T7 G7 G T T9 C12 A12 G12 G12 G12 C12

>AF103818_SIV-CPZ-US 25 A A11 A11 A C11 T11 G11 C11 T11 G11 A11 C T11 G A G19 G19 A18 C18 T18 T18 T18 C18 T10 A26
A26 G17 G17 G17 A15 C15 T15 T15 T15 C15 C15 A15
A17 G17 G17 G17 A17 C17 G T T C C A
A G G G G11 G11

>X52154_SIV-CPZ-GAB 27 A C11 T11 G11 C11 T11 G11 A11 C T11
C T G C G17 G17 G17 A15 C15 T15 T15 T15 C15 C15 A15
A17 G17 G17 G17 A17 C17 T T T C C G G G G A13 G13 A

>AJ271370_HIV-1-N 1 C13 A13 A13 G13 G13 G13 A13 C13 T13 T13 T13
A13 C11 A11 C15 G15 G18 G18 G18 A18 C18 T18 T18 T18 C18 C18 G15
C18 C18 A18 G G G G C T T T C C A G G A10 A G G

>AJ006022_HIV-1-N 1 C13 A13 A13 G13 G13 G13 A13 C13 T13 T13 T13
A13 C11 T11 G11 C11 T11 G11 A11 C15 A17 A18 G19 G19 G19 A18 C18 T18 T18 T18
A T9 A9 C9 T9 T G15 G18 G18 G18 A18 C18 T18 T18 T18 C18 C18 G15
C18 C18 A18 G21 G21 G21 A18 C18 T18 T18 T18 C18 C18 A15 G23 G23 G23 A13 G13 G13

```
>L20587_HIV-1-O     18 A13 T9 T18 G18 C18 T18 G18 A18 C18 A18 C18 T18 G18 T G G A A C T T T C C A G C11 A11 A10 A15 G14
                       A14 C14 T29 G29 C29 T29 G29 A29 C29 A29 C29 T29 G17 C17 G17 G17 G17 G17 A17 C17 T17 T17 T17 C17 C17 A17 G17 T17 G17 G16 G16 A16

>L20571_HIV-1-O     1 C18 T18 G18 A18 C18 A18 C18 T18 G18 C22 G22 G22 G22 A15 C15 T15 T15 T15 C15 C15 A15 G14
                       A14 C14 T29 G29 C29 T29 G29 A29 C29 A29 C29 T29 G17 C17 G17 G17 G17 G17 A17 C17 T17 T17 T17 C17 C17 A17 G17 C11 G T G G

>AJ302647_HIV-1-O   17 A13 T9 T18 G18 C18 T18 G18 A18 C18 A18 C18 T18 G18 C22 G22 G22 G22 A15 C15 T15 T15 T15 C15 C15 A15 G14 C11 A11 G10 A10 G10
                       G10 C T G C T G A C A C G G17 C17 G17 G17 G17 G17 A17 C17 T17 T17 T17 C17 C17 A17 G17 T17 G17 T G G16

>L07625_HIV-2-B     93 T14 A G C T G A C A C C8 G36 C36 A36 G36 G15 G15 A15 C15 T15 T15 T15 C15 C15 A15 A36 A36 A36 G36 A36 G10

>X61240_HIV-2-B     54 T A G C A G A C A17 C T G36 C36 A36 G36 G15 G15 A15 C15 T15 T15 T15 C15 C15 A15 A36 A36 A36 G36 A36 G10

>M19499_SIV-SMM-MAC 41 T14 C G C T G A G A T9 A17 G17 C17 A17 G17 G15 G15 A15 C15 T15 T15 T15 C15 C15 A15 C22 A22 A10 G10 G10 G10

>X14307_SIV-SMM     40 A9 A G C T G10 A10 G9 A17 C17 A17 G17 C17 A17 G17 G15 G15 A15 C15 T15 T15 T15 C15 C15 A15 C22 A22 A10 G10 G10 G10

>AF208027_HIV-2-G   14 A9 A G C T G A C A17 C17 A17 G17 C17 A17 G17 G15 G15 A15 C15 T15 T15 T15 C15 C15 A15 G15 T A T G10 G10

>M30895_HIV-2-A     49 C17 A17 G17 C17 T17 G17 A17 G9 G C T G C A
                       G92 C41 T41 G41 C41 A41 G41 G15 G15 A15 C15 T15 T15 T15 C15 C15 A15 G15 A41 A10 G10 G10 G10

>J04542_HIV-2-A     46 C17 A17 G17 C17 T17 G17 A17 G9 A17 C41 T41 G41 C41 A41 G41 G15 G15 A15 C15 T15 T15 T15 C15 C15 A15 G15 A41 A10 G10 G10 G10

>M15390_HIV-2-A     49 C17 A17 G17 C17 T17 G17 A17 G9 A17 C41 T41 G41 C41 A41 G41 G15 G15 A15 C15 T15 T15 T15 C15 C15 A15 G15 A41 A10 G10 G10 G10

>M31113_HIV-2-A     50 C17 A17 G17 C17 T17 G17 A17 G9 A17 C41 T41 G41 C41 A41 G41 G15 G15 A15 C15 T15 T15 T15 C15 C15 A15 G15 A41 A10 G10 G10 G10

>D00835_HIV-2-A     49 C17 A17 G17 C17 T17 G17 A17 G9 A17 C41 T41 G41 C41 A41 G41 G15 G15 A15 C15 T15 T15 T15 C15 C15 A15 G15 A41 A10 G10 G10 G10

>J04498_HIV-2-A     49 C17 A17 G17 C17 T17 G17 A17 G9 A17 C41 T41 G41 C41 A41 G41 G15 G15 A15 C15 T15 T15 T15 C15 C15 A15 G15 A41 A10 G10 G10 G10

>J03654_HIV-2-A     49 C17 A17 G17 C17 T17 G17 A17 G9 A17 C41 T41 G41 C41 A41 G41 G15 G15 A15 C15 T15 T15 T15 C15 C15 A15 G15 A41 A10 G10 G10 G10

>M30502_HIV-2-A     47 C17 A17 G17 C17 T17 G17 A17 G9 G92 C41 T41 G41 C41 A41 G41 G15 G15 A15 C15 T15 T15 T15 C15 C15 A15 G15 A41 A10 G10 G10 G10
```
